# Crystal structure of olivetolic acid: a natural product from *Cetrelia sanguinea* (Schaer.)

**DOI:** 10.1107/S2056989016016273

**Published:** 2016-10-18

**Authors:** Friardi Ismed, Aulia Farhan, Amri Bakhtiar, Erizal Zaini, Yuda Prasetya Nugraha, Okky Dwichandra Putra, Hidehiro Uekusa

**Affiliations:** a The Laboratory of Natural Resource of Sumatra (LBS) and Faculty of Pharmacy, Andalas University, 26163 Padang, Indonesia; b Department of Chemistry and Materials Science, Tokyo Institute of Technology, Ookayama 2-12-1, Meguro-ku, Tokyo 152-8551, Japan

**Keywords:** crystal structure, olivetolic acid, *Cetrelia sanguinea*

## Abstract

The title compound, C_12_H_16_O_4_ (systematic name: 2,4-dihy­droxy-6-pentyl­benzoic acid) is a natural product isolated from *C. sanguinea* (Schaer.) and is reported to have various pharmacological activities. The mol­ecule is approximately planar (r.m.s. deviation for the non-H atoms = 0.096 Å) and features an intra­molecular O—H⋯O hydrogen bond. In the crystal, each olivetolic acid mol­ecule is connected to three neighbours *via* O—H⋯O hydrogen bonds, generating (10-1) sheets. This crystal is essentially isostructural with a related resorcinolic acid with a longer alkyl chain.

## Abstract 2

The packing in olivetolic acid is similar to that in resorcinolic acid.

## Chemical context   

Monoaromatic compounds from lichens have attracted a great inter­est in the pharmaceutical field due to their potential pharmacological activities such as anti­bacterial, anti­fungal, cytotoxic, and photoprotective activities (Gianini *et al.*,2008 ▸: Stocker-Wörgötter, 2008 ▸; Ismed *et al.*, 2012 ▸). The title compound, C_12_H_16_O_4_, is a derivative of alkyl resorcinolic acid which is commonly found in certain species of lichens (Gomes *et al.*, 2006 ▸).
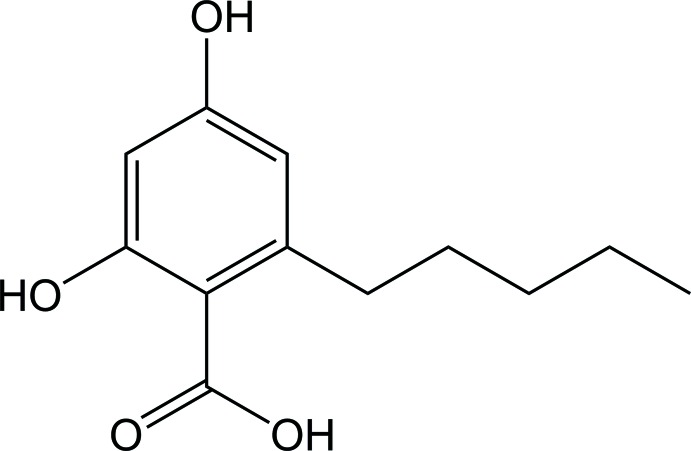


## Structural commentary   

**Figure 1:**
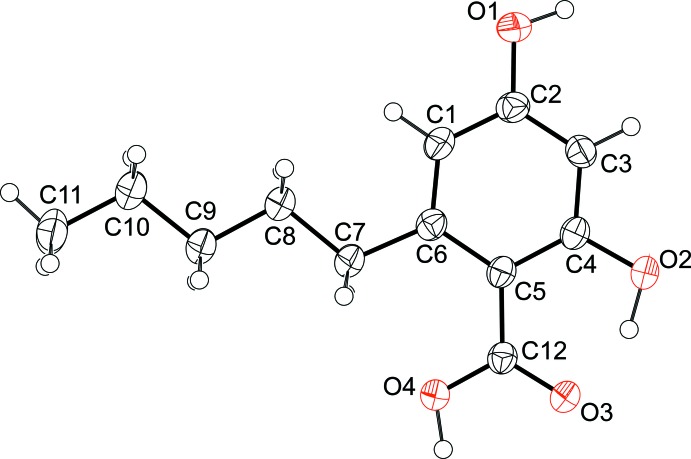
The mol­ecular structure of the title compound, showing 50% probability displacement ellipsoids.

The title compound (Fig. 1 ▸) crystallizes with monoclinic metric symmetry and adopts a roughly planar conformation (r.m.s. deviation = 0.093 Å). All bond distances, angles and dihedral angles appear to be usual except the bond angle of C6—C5—C12 [124.61 (13)°] compared to the mean value and their standard deviation of selected 24 similar structures reported in Cambridge Structural Database (CSD, Version 5.37, Update 2 Feb 2016; Groom *et al.*, 2016 ▸). In this case, the deviating bond angle may be a result of the strong intra­molecular O2—H2⋯O3 inter­action.

## Supra­molecular features   

**Figure 2:**
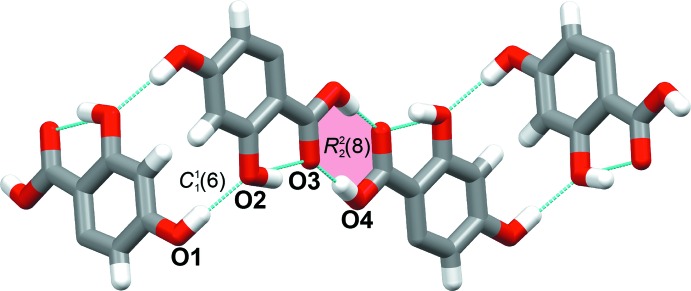
A partial view of the packing in the title compound, showing the hydrogen-bonded chain structure, formed through O—H⋯O hydrogen bonds. Blue dashed lines indicate hydrogen bonds.

In the crystal, each mol­ecule is connected with three others (Fig. 2 ▸): O1 acts as an O—H⋯O hydrogen bond donor while O2 is an O—H⋯O acceptor, forming a 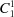
(6) infinite chain. In addition, an O4—H4⋯O3 carb­oxy­lic acid homodimer synthon is observed, generating an 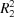
(8) loop. Together, these hydrogen bonds construct a layered architecture propagating in the (10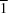) plane. Details of the hydrogen bonds are given in Table 1 ▸.

**Figure 3:**
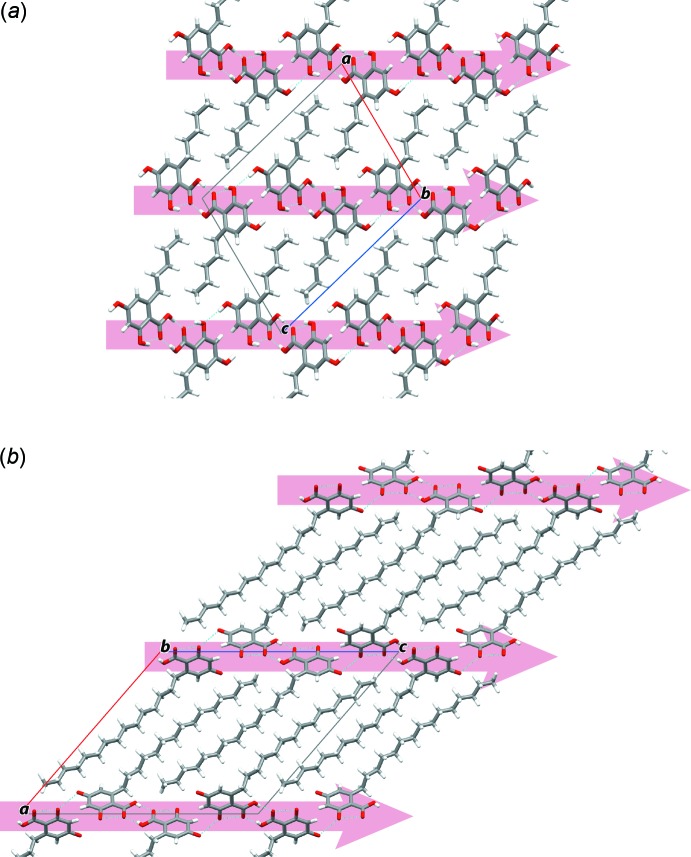
Crystal-packing views along *b* axis of (*a*) the title compound and (*b*) 6-*n*-penta­decyl-2,4-di­hydroxy­benzoic acid. Both structures possess isostructurality. The arrows indicate the one-dimensional hydrogen-bond chains involving resorsinolic acid.

Inter­estingly, the title compound showed isostructurality with alkyl resorsinolic acid derivatives with longer alkyl chain of 6-*n*-penta­decyl-2,4-dihy­droxy-benzoic acid (Gadret *et al.*, 1975 ▸; refcode: PDCHBZ10). Both structures exhibited extremely similar hydrogen bond in resorsinolic acid shown in Fig. 3 ▸ *a* and 3*b*. Both crystal structures consist of a hydro­philic layer of the resorcinol acid moiety with hydrogen-bonding inter­actions, and a hydro­phobic layer of normal alkyl chains.

## Crystallization   

Crystallization of the title compound was conducted by dissolving 700 mg of the isolate in an ethyl acetate–hexane solvent mixture (1:1). The solution was kept for one week at room temperature yielding colourless needles of the title compound.

## Refinement   

Crystal data, data collection and structure refinement details are summarized in Table 2 ▸. All non-hydrogen atoms were refined anistropically. The hydrogen atoms of O hy­droxy and O carb­oxy­lic acid were located from a difference Fourier map and were refined isotropically. All other hydrogen atoms were located geometrically and refined as riding [*U*_iso_ = 1.5*U*_iso_(C) for the terminal alkyl group and *U*_iso_ = 1.2*U*_iso_(C) for other hydrogen atoms].

## Supplementary Material

Crystal structure: contains datablock(s) I. DOI: 10.1107/S2056989016016273/hb7614sup1.cif

Structure factors: contains datablock(s) I. DOI: 10.1107/S2056989016016273/hb7614Isup2.hkl

Supporting information file. DOI: 10.1107/S2056989016016273/hb7614Isup3.cml

CCDC reference: 1509626

Additional supporting information: crystallographic information; 3D view; checkCIF report

## Figures and Tables

**Table 1:** Hydrogen-bond geometry (Å, °)

*D*—H⋯*A*	*D*—H	H⋯*A*	*D*⋯*A*	*D*—H⋯*A*
O1—H1*A*⋯O2^i^	0.93 (2)	1.90 (2)	2.8168 (16)	169.6 (19)
O2—H2⋯O3	1.00 (3)	1.58 (3)	2.5043 (14)	152 (2)
O4—H4⋯O3^ii^	0.94 (3)	1.70 (3)	2.6368 (15)	177 (2)

Symmetry codes: (i) 
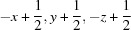
; (ii) 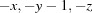.

**Table 2:** Experimental details

Crystal data	
Chemical formula	C_12_H_16_O_4_
*M*_r_	224.25
Crystal system, space group	Monoclinic, *P*2_1_/*n*
Temperature (K)	173
*a*, *b*, *c* (Å)	14.2527 (8), 4.7524 (3), 17.6489 (11)
β (°)	103.538 (4)
*V* (Å^3^)	1162.22 (12)
*Z*	4
Radiation type	Cu *K*α
μ (mm^−1^)	0.79
Crystal size (mm)	0.12 × 0.10 × 0.10

Data collection	
Diffractometer	RIGAKU R-AXIS RAPID II
Absorption correction	Multi-scan (*ABSCOR*; Higashi, 1995 ▸)
*T*_min_, *T*_max_	0.789, 0.924
No. of measured, independent and observed [*I* > 2σ(*I*)] reflections	12627, 2087, 1762
*R*_int_	0.036
(sin θ/λ)_max_ (Å^−1^)	0.602

Refinement	
*R*[*F* ^2^ > 2σ(*F* ^2^)], *wR*(*F* ^2^), *S*	0.045, 0.136, 1.14
No. of reflections	2087
No. of parameters	158
H-atom treatment	H atoms treated by a mixture of independent and constrained refinement
Δρ_max_, Δρ_min_ (e Å^−3^)	0.29, −0.18

Computer programs: *PROCESS-AUTO* (Rigaku, 1998 ▸), *SHELXS2014* (Sheldrick, 2008 ▸) and *SHELXL2014* (Sheldrick, 2015 ▸).

## supplementary crystallographic information

### Crystal data

**Table:**

C_12_H_16_O_4_	*F*(000) = 480
*M**_r_* = 224.25	*D*_x_ = 1.282 Mg m^−^^3^
Monoclinic, *P*2_1_/*n*	Cu *K*α radiation, λ = 1.54186 Å
*a* = 14.2527 (8) Å	Cell parameters from 12628 reflections
*b* = 4.7524 (3) Å	θ = 3.6–68.2°
*c* = 17.6489 (11) Å	µ = 0.79 mm^−^^1^
β = 103.538 (4)°	*T* = 173 K
*V* = 1162.22 (12) Å^3^	Block, colorless
*Z* = 4	0.12 × 0.10 × 0.10 mm

### Data collection

**Table:**

RIGAKU R-AXIS RAPID II diffractometer	2087 independent reflections
Radiation source: rotating anode X-ray	1762 reflections with *I* > 2σ(*I*)
Detector resolution: 10.0 pixels mm^-1^	*R*_int_ = 0.036
ω–scan	θ_max_ = 68.2°, θ_min_ = 3.6°
Absorption correction: multi-scan (ABSCOR; Higashi, 1995)	*h* = −17→17
*T*_min_ = 0.789, *T*_max_ = 0.924	*k* = −5→5
12627 measured reflections	*l* = −20→21

### Refinement

**Table:**

Refinement on *F*^2^	Primary atom site location: structure-invariant direct methods
Least-squares matrix: full	Secondary atom site location: none
*R*[*F*^2^ > 2σ(*F*^2^)] = 0.045	Hydrogen site location: mixed
*wR*(*F*^2^) = 0.136	H atoms treated by a mixture of independent and constrained refinement
*S* = 1.14	*w* = 1/[σ^2^(*F*_o_^2^) + (0.0808*P*)^2^ + 0.1676*P*] where *P* = (*F*_o_^2^ + 2*F*_c_^2^)/3
2087 reflections	(Δ/σ)_max_ < 0.001
158 parameters	Δρ_max_ = 0.29 e Å^−^^3^
0 restraints	Δρ_min_ = −0.18 e Å^−^^3^

### Special details

**Table:**

Geometry. All esds (except the esd in the dihedral angle between two l.s. planes) are estimated using the full covariance matrix. The cell esds are taken into account individually in the estimation of esds in distances, angles and torsion angles; correlations between esds in cell parameters are only used when they are defined by crystal symmetry. An approximate (isotropic) treatment of cell esds is used for estimating esds involving l.s. planes.

### Fractional atomic coordinates and isotropic or equivalent isotropic displacement parameters (Å^2^)

**Table:**

	*x*	*y*	*z*	*U*_iso_*/*U*_eq_	
O1	0.38939 (8)	0.5564 (2)	0.16723 (7)	0.0419 (3)	
C1	0.32068 (11)	0.2528 (3)	0.06446 (9)	0.0333 (4)	
H1	0.3726	0.2983	0.0413	0.040*	
H1A	0.3901 (15)	0.588 (4)	0.2194 (14)	0.060 (6)*	
O2	0.09864 (8)	0.0870 (2)	0.17140 (6)	0.0348 (3)	
H2	0.0574 (18)	−0.059 (5)	0.1389 (14)	0.079 (7)*	
C2	0.31656 (11)	0.3787 (3)	0.13488 (9)	0.0321 (4)	
C3	0.24081 (11)	0.3235 (3)	0.16913 (9)	0.0317 (4)	
H3	0.2370	0.4140	0.2163	0.038*	
O3	0.03156 (7)	−0.2603 (2)	0.06590 (6)	0.0345 (3)	
C4	0.17037 (10)	0.1334 (3)	0.13338 (8)	0.0286 (4)	
O4	0.10317 (8)	−0.3497 (2)	−0.03022 (6)	0.0378 (3)	
H4	0.0556 (18)	−0.491 (6)	−0.0410 (14)	0.080 (7)*	
C5	0.17377 (10)	−0.0040 (3)	0.06311 (8)	0.0275 (3)	
C6	0.25149 (10)	0.0639 (3)	0.02743 (8)	0.0287 (4)	
C7	0.25914 (11)	−0.0625 (3)	−0.04994 (9)	0.0336 (4)	
H7A	0.1981	−0.0241	−0.0887	0.040*	
H7B	0.2648	−0.2692	−0.0435	0.040*	
C8	0.34233 (11)	0.0400 (3)	−0.08352 (9)	0.0368 (4)	
H8A	0.3396	0.2476	−0.0879	0.044*	
H8B	0.4042	−0.0109	−0.0474	0.044*	
C9	0.33918 (12)	−0.0863 (3)	−0.16340 (9)	0.0374 (4)	
H9A	0.3426	−0.2939	−0.1587	0.045*	
H9B	0.2768	−0.0377	−0.1992	0.045*	
C10	0.42080 (13)	0.0161 (4)	−0.19841 (10)	0.0454 (5)	
H10A	0.4185	0.2240	−0.2015	0.054*	
H10B	0.4831	−0.0373	−0.1632	0.054*	
C11	0.41710 (14)	−0.1014 (4)	−0.27894 (11)	0.0516 (5)	
H11A	0.4215	−0.3071	−0.2762	0.077*	
H11B	0.4713	−0.0265	−0.2982	0.077*	
H11C	0.3562	−0.0466	−0.3145	0.077*	
C12	0.09866 (10)	−0.2112 (3)	0.03296 (8)	0.0283 (3)	

### Atomic displacement parameters (Å^2^)

**Table:**

	*U*^11^	*U*^22^	*U*^33^	*U*^12^	*U*^13^	*U*^23^
O1	0.0439 (6)	0.0464 (7)	0.0368 (7)	−0.0190 (5)	0.0123 (5)	−0.0070 (5)
C1	0.0333 (8)	0.0361 (8)	0.0328 (8)	−0.0048 (6)	0.0128 (6)	0.0029 (6)
O2	0.0348 (6)	0.0417 (6)	0.0319 (6)	−0.0064 (5)	0.0160 (5)	−0.0050 (5)
C2	0.0332 (7)	0.0317 (7)	0.0307 (8)	−0.0051 (6)	0.0061 (6)	0.0033 (6)
C3	0.0372 (8)	0.0312 (8)	0.0270 (8)	−0.0014 (6)	0.0081 (7)	−0.0017 (6)
O3	0.0350 (6)	0.0378 (6)	0.0342 (6)	−0.0084 (4)	0.0151 (5)	−0.0036 (5)
C4	0.0300 (7)	0.0292 (7)	0.0278 (8)	0.0010 (6)	0.0094 (6)	0.0048 (6)
O4	0.0409 (6)	0.0421 (6)	0.0350 (6)	−0.0140 (5)	0.0182 (5)	−0.0108 (5)
C5	0.0297 (7)	0.0273 (7)	0.0267 (8)	−0.0001 (6)	0.0089 (6)	0.0041 (6)
C6	0.0309 (7)	0.0285 (7)	0.0271 (8)	−0.0006 (6)	0.0076 (6)	0.0054 (6)
C7	0.0363 (8)	0.0352 (8)	0.0327 (9)	−0.0053 (6)	0.0148 (7)	0.0004 (6)
C8	0.0381 (8)	0.0414 (9)	0.0348 (9)	−0.0073 (7)	0.0161 (7)	−0.0018 (7)
C9	0.0399 (8)	0.0406 (9)	0.0362 (9)	−0.0048 (7)	0.0179 (7)	−0.0009 (7)
C10	0.0467 (9)	0.0509 (10)	0.0457 (10)	−0.0076 (8)	0.0253 (8)	−0.0038 (8)
C11	0.0581 (11)	0.0594 (11)	0.0462 (11)	−0.0007 (9)	0.0299 (9)	0.0023 (9)
C12	0.0310 (7)	0.0279 (7)	0.0271 (7)	−0.0004 (6)	0.0090 (6)	0.0034 (6)

### Geometric parameters (Å, º)

**Table:**

O1—C2	1.3563 (18)	C6—C7	1.519 (2)
O1—H1A	0.93 (2)	C7—C8	1.5243 (19)
C1—C6	1.380 (2)	C7—H7A	0.9900
C1—C2	1.393 (2)	C7—H7B	0.9900
C1—H1	0.9500	C8—C9	1.523 (2)
O2—C4	1.3657 (16)	C8—H8A	0.9900
O2—H2	1.00 (3)	C8—H8B	0.9900
C2—C3	1.380 (2)	C9—C10	1.519 (2)
C3—C4	1.388 (2)	C9—H9A	0.9900
C3—H3	0.9500	C9—H9B	0.9900
O3—C12	1.2520 (16)	C10—C11	1.516 (2)
C4—C5	1.412 (2)	C10—H10A	0.9900
O4—C12	1.3094 (17)	C10—H10B	0.9900
O4—H4	0.94 (3)	C11—H11A	0.9800
C5—C6	1.4333 (19)	C11—H11B	0.9800
C5—C12	1.460 (2)	C11—H11C	0.9800
			
C2—O1—H1A	110.4 (13)	C9—C8—C7	112.14 (13)
C6—C1—C2	121.82 (13)	C9—C8—H8A	109.2
C6—C1—H1	119.1	C7—C8—H8A	109.2
C2—C1—H1	119.1	C9—C8—H8B	109.2
C4—O2—H2	103.8 (14)	C7—C8—H8B	109.2
O1—C2—C3	122.28 (14)	H8A—C8—H8B	107.9
O1—C2—C1	117.06 (13)	C10—C9—C8	113.00 (13)
C3—C2—C1	120.66 (14)	C10—C9—H9A	109.0
C2—C3—C4	118.78 (14)	C8—C9—H9A	109.0
C2—C3—H3	120.6	C10—C9—H9B	109.0
C4—C3—H3	120.6	C8—C9—H9B	109.0
O2—C4—C3	115.27 (13)	H9A—C9—H9B	107.8
O2—C4—C5	122.70 (13)	C11—C10—C9	113.66 (15)
C3—C4—C5	122.02 (13)	C11—C10—H10A	108.8
C12—O4—H4	110.8 (15)	C9—C10—H10A	108.8
C4—C5—C6	118.06 (13)	C11—C10—H10B	108.8
C4—C5—C12	117.30 (12)	C9—C10—H10B	108.8
C6—C5—C12	124.61 (13)	H10A—C10—H10B	107.7
C1—C6—C5	118.60 (13)	C10—C11—H11A	109.5
C1—C6—C7	119.31 (13)	C10—C11—H11B	109.5
C5—C6—C7	122.09 (13)	H11A—C11—H11B	109.5
C6—C7—C8	116.69 (13)	C10—C11—H11C	109.5
C6—C7—H7A	108.1	H11A—C11—H11C	109.5
C8—C7—H7A	108.1	H11B—C11—H11C	109.5
C6—C7—H7B	108.1	O3—C12—O4	119.78 (13)
C8—C7—H7B	108.1	O3—C12—C5	122.09 (13)
H7A—C7—H7B	107.3	O4—C12—C5	118.12 (12)
			
C6—C1—C2—O1	178.54 (13)	C12—C5—C6—C1	−176.44 (13)
C6—C1—C2—C3	−1.8 (2)	C4—C5—C6—C7	−177.26 (12)
O1—C2—C3—C4	−178.34 (14)	C12—C5—C6—C7	4.3 (2)
C1—C2—C3—C4	2.0 (2)	C1—C6—C7—C8	−2.5 (2)
C2—C3—C4—O2	179.08 (12)	C5—C6—C7—C8	176.74 (13)
C2—C3—C4—C5	−0.2 (2)	C6—C7—C8—C9	−176.33 (13)
O2—C4—C5—C6	178.97 (12)	C7—C8—C9—C10	179.26 (14)
C3—C4—C5—C6	−1.8 (2)	C8—C9—C10—C11	−178.38 (15)
O2—C4—C5—C12	−2.5 (2)	C4—C5—C12—O3	2.4 (2)
C3—C4—C5—C12	176.76 (13)	C6—C5—C12—O3	−179.11 (13)
C2—C1—C6—C5	−0.3 (2)	C4—C5—C12—O4	−176.99 (12)
C2—C1—C6—C7	179.01 (13)	C6—C5—C12—O4	1.5 (2)
C4—C5—C6—C1	2.0 (2)		

### Hydrogen-bond geometry (Å, º)

**Table:**

*D*—H···*A*	*D*—H	H···*A*	*D*···*A*	*D*—H···*A*
O1—H1*A*···O2^i^	0.93 (2)	1.90 (2)	2.8168 (16)	169.6 (19)
O2—H2···O3	1.00 (3)	1.58 (3)	2.5043 (14)	152 (2)
O4—H4···O3^ii^	0.94 (3)	1.70 (3)	2.6368 (15)	177 (2)

Symmetry codes: (i) −*x*+1/2, *y*+1/2, −*z*+1/2; (ii) −*x*, −*y*−1, −*z*.
